# P-2173. Evaluation of Self/Parent Collected Oral/Nasal Swabs for Respiratory Illness in Symptomatic Children

**DOI:** 10.1093/ofid/ofae631.2327

**Published:** 2025-01-29

**Authors:** Hannah Nelson, Iryna Kayda, Nicole Watson, Maggie Woo Kinshella, Karen Mooder, Jennifer Cochrane, Neil Desai, Linda Hoang, Michelle Dittrick, David M Goldfarb

**Affiliations:** University of British Columbia, Vancouver, British Columbia, Canada; The University of British Columbia, Surrey, British Columbia, Canada; BC Children’s and Women’s Hospital and Health Centre, Vancouver, British Columbia, Canada; University of British Columbia, Vancouver, British Columbia, Canada; First Nations Health Authority, West Vancouver, British Columbia, Canada; First Nation Health Authority, Vancouver, British Columbia, Canada; University of British Columbia, Vancouver, British Columbia, Canada; British Columbia Center for Disease Control, Vancouver, BC, Canada; BC Children's Hospital, Vancouver, British Columbia, Canada; University of British Columbia, Vancouver, British Columbia, Canada

## Abstract

**Background:**

For children under the age of 4 who are unable to reliably gargle, a combined oral/nasal (ON) swab collected by the parent/caregiver may be offered, and is less invasive than the standard nasopharyngeal (NP) swab. We sought to determine the diagnostic yield and acceptability of self-collected ON swab samples compared to healthcare worker-collected NP swabs for respiratory viruses, including severe acute respiratory syndrome coronavirus 2 (SARS-CoV-2), influenza, and respiratory syncytial virus (RSV) in children.

**Methods:**

Symptomatic participants (0-4 years old) were recruited from a pediatric Emergency Department (ED) where they were undergoing clinical testing with an NP swab. Participants provided an additional ON swab using written self-collection instructions. Caregivers were asked to rate acceptability of ON and NP swab collections on a 5-point Likert scale. Both samples were collected with minitip flocked swabs and were tested with the SARS-CoV-2/Influenza A+B/RSV GeneXpert® assay. A portion of matched samples were also tested with the BioFire® RP2.1 respiratory panel.

**Results:**

A total of 121 matched sample pairs were tested with the GeneXpert assay and had similar detection for RSV (28 ON positive vs 26 NP positive), influenza A (17 ON positive vs 17 NP positive), and SARS-CoV-2 (8 ON positive vs 10 NP positive). Median Ct values were higher for matched ON positives vs NP positives (26.2 vs 23.9). 58 pairs were also tested using the RP2.1 assay and showed a similar yield for total respiratory viruses detected (63 positive ON swabs vs 62 positive NP swabs). With either sample positive set as the reference for calculation of sensitivity, the ON and NP swab sensitivities are as follows: RSV (80.0% ON vs 100.0% NP), influenza A (94.4% ON vs 94.4% NP), SARS-CoV-2 (100.0% ON and 92.9% NP), and all other virus targets (82.6% ON vs 82.6% NP). Caregivers rated higher acceptability for ON swabs than HCW-collected NP swabs (median acceptability 2 vs 4).

**Conclusion:**

The performance of self-collected ON swabs was similar to HCW-collected NP swabs for the detection of a range of respiratory viruses across two commercial molecular assays. Given their significantly higher acceptability ratings, self-collected ON swabs should be considered as a potential less-invasive diagnostic option for children.

**Disclosures:**

All Authors: No reported disclosures

